# Current Status of COVID-19 Therapies and Drug Repositioning Applications

**DOI:** 10.1016/j.isci.2020.101303

**Published:** 2020-06-20

**Authors:** Ozlem Altay, Elyas Mohammadi, Simon Lam, Hasan Turkez, Jan Boren, Jens Nielsen, Mathias Uhlen, Adil Mardinoglu

**Affiliations:** 1 Science for Life Laboratory, KTH – Royal Institute of Technology, Stockholm 17121, Sweden; 2 Department of Animal Science, Ferdowsi University of Mashhad, Mashhad 9177948974, Iran; 3 Centre for Host-Microbiome Interactions, Faculty of Dentistry, Oral & Craniofacial Sciences, King's College London, London SE1 9RT, UK; 4 Department of Medical Biology, Faculty of Medicine, Atatürk University, Erzurum 25240, Turkey; 5 Department of Molecular and Clinical Medicine, University of Gothenburg, The Wallenberg Laboratory, Sahlgrenska University Hospital, Gothenburg 41345, Sweden; 6 Department of Biology and Biological Engineering, Chalmers University of Technology, SE-Gothenburg, 41296, Sweden

**Keywords:** Health Sciences, Medicine

## Abstract

The rapid and global spread of a new human coronavirus (SARS-CoV-2) has produced an immediate urgency to discover promising targets for the treatment of COVID-19. Drug repositioning is an attractive approach that can facilitate the drug discovery process by repurposing existing pharmaceuticals to treat illnesses other than their primary indications. Here, we review current information concerning the global health issue of COVID-19 including promising approved drugs and ongoing clinical trials for prospective treatment options. In addition, we describe computational approaches to be used in drug repurposing and highlight examples of *in silico* studies of drug development efforts against SARS-CoV-2.

## Introduction

**Figure 1:**
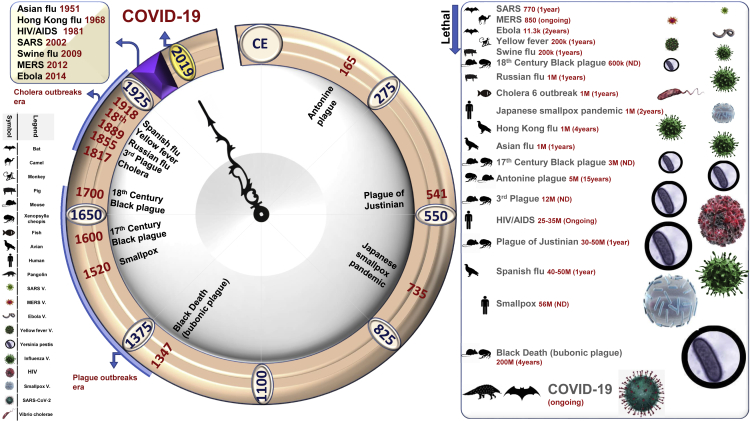
Deadliest Pandemics and Outbreaks in Recorded History Left: Pandemic clock, a comprehensive illustration of the most deadly pandemics through the history sorted by date. Right: Pandemic outbreaks and natural reservoir hosts sorted by the number of mortalities. In some outbreaks, the exact duration is not precisely determined (ND) because the outbreak died out and recurred multiple times in the same region and era.

Over the past few centuries, outbreaks caused by bacteria like *Yersinia pestis* and *Vibrio cholerae* (the causative agents of plague and cholera, respectively, Cohn and Kutalek, 2016; Pechous et al., 2016) and also by viral infectious agents such as influenza viruses, Ebola viruses, SARS-CoV-1, MERS-CoV, the Zika virus, and lately, SARS-CoV-2, have undermined public trust in the ability of modern science to predict and prevent global pandemic threats. Accordingly, studying historical epidemics can help us identify patterns of viral outbreaks and design a plan to prepare for the next pandemic. For example, if the unusual influenza-like illness from February to August 1918 was considered a harbinger, then perhaps the 1918 Spanish flu pandemic could have resulted in fewer casualties (Simonsen et al., 2018). An overview of the deadliest historical pandemics from Antonine Plague in 165 CE to the ongoing COVID-19 (coronavirus disease-2019) pandemic, based on data from the World Health Organization (WHO) and US Centers for Disease Control and Prevention, is illustrated in Figure 1 and includes the dates, natural reservoir host, and the number of mortalities.

In December 2019, rumors began to spread about the prevalence of a new unknown pneumonia-like illness in Wuhan, the capital of Hubei Province in the People's Republic of China. Soon afterward, authorities reported a novel coronavirus as the causative agent of clusters of the new illness (WHO, February 11, 2020). COVID-19 was the name that the WHO designated for the disease caused by the novel coronavirus, designated 2019-nCoV and later severe acute respiratory syndrome coronavirus 2 (SARS-CoV-2) (Gorbalenya, 2020). Since the beginning of the outbreak, infections have expanded rapidly into multiple simultaneous epidemics worldwide. At the time of writing, over 7.2 million confirmed COVID-19 cases and over 410,000 COVID-19-related deaths have been reported across more than 180 countries (https://coronavirus.jhu.edu/map.html).

The geographical distribution of COVID-19 covers all continents except Antarctica. Epidemiologists in Wuhan believe the Huanan Seafood Wholesale Market in Wuhan to be the point of origin of SARS-CoV-2, due to its connection to the trading of live wild animals (WHO January 22, 2020) (Zhou et al., 2020a). COVID-19 is highly contagious, with a basic reproduction number (*R*_0_) of between 1.4 and 6.5, and can be easily spread through coughs and sneezes, talking to infected persons, and touching the eyes, nose, or mouth after touching a contaminated surface (Liu et al., 2020).

SARS-CoV-2 has been found in stool and blood samples (Chen et al., 2020b; Tang et al., 2020), but the rate of transmission (Chan et al., 2020; Rothe et al., 2020), period of infectivity (Zou et al., 2020), and duration of viral shedding (Zhou et al., 2020a) are various and uncertain. Large-scale serologic screening provides a better picture of statistics related to asymptomatic infections for epidemiologic analysis.

The phylogeny, virology, and epidemiology of SARS-CoV-2 is being studied extensively. At the genome level, SARS-CoV-2 has 79.5% homology to SARS-CoV-1, the causative agent of SARS in south China; 85%–96% identity with bat-SL-CoVZC45 (Li et al., 2005), a bat SARS-like coronavirus (Xiao et al., 2020); and 91.02% similarity with SARS-CoV-2-like coronavirus in pangolin named Pangolin-CoV (Yu et al., 2020). A population genetic analysis of 103 SARS-CoV-2 genomes from China revealed that the causative agent of COVID-19 consists of two evolution types. Type L (70% prevalence in Wuhan), which is derived from the ancestral type S (30% prevalence in Wuhan), is more aggressive and contagious (Tang et al., 2020b). Zhou et al. investigated the COVID-19 agent through full genome sequencing and phylogenetic analysis, classifying it as a betacoronavirus (a positive-sense, single-stranded RNA virus), the same subgenus as SARS-CoV as well as several bat coronaviruses (Zhou et al., 2020b). In addition, they proved that SARS-CoV-2 uses the same receptor binding (angiotensin-converting enzyme 2 [ACE2]) and cell entry pathway as SARS-CoV-1. When compared with SARS-CoV-1, MERS-CoV is less closely related to SARS-CoV-2 (Lu et al., 2020; Zhu et al., 2020).

Taken together, there is an urgent need for COVID-19 therapeutics due to the S-shaped curve expansion of the infections, widespread pandemic status, and global burden. Given the similarities between SARS-CoV-2 and other coronaviruses, and its relative ease of sample acquisition and study, it has been widely accepted that drug repositioning is a promising approach to make available an effective, safety-assured treatment in a timely manner. In this review, we summarize diagnostic approaches, risk groups, available treatment options, and drug repositioning studies related to COVID-19.

## Diagnostic Approaches and Medical Comorbidities

Before efficient treatment, precise diagnosis and classification of patients based on disease severity and their probable vulnerability to COVID-19 is crucial. Diagnosis has several steps that can be prioritized based on the provision of facilities during an outbreak. Generally, evaluation of symptoms and particular laboratory features that are associated with worse cases come first. Second, laboratory detection as a complementary test is done to confirm the infection. Clinical manifestations can help to classify patients and categorize efficient remedies for each cohort.

At the beginning, due to the unknown nature of this emergent virus, all diagnosis was based purely on clinical and radiographic findings (i.e., bilateral infiltrates on chest imaging), failure of antibiotic therapy, and exclusion of other common respiratory infections (Huang et al., 2020; Zhu et al., 2020). Now, laboratory detection includes next-generation sequencing platforms, reverse-transcription polymerase chain reaction (RT-PCR), and serological methods like enzyme-linked immunoassay. Generally, a patient should be taken into account for laboratory detection if his or her clinical manifestations and/or radiographical records are positive. From the most common symptoms to the least—these are not constant—for COVID-19 fever, fatigue, dry cough, anorexia, myalgias, dyspnea, and sputum production are known so far (Wang et al., 2020b). Recently, anosmia and dysgeusia have been added to this list as common symptoms (Giacomelli et al., 2020). In addition, some less common complaints are headache, sore throat, rhinorrhea, and gastrointestinal disorders (e.g., nausea and diarrhea) (Chen et al., 2020a).

Another procedure that is being used to confirm the infection is monitoring laboratory features that are associated with worse outcomes. A positive correlation has been reported in worse cases between COVID-19 and sex (the rate of death for males was significantly more than that for females in China and Italy) (Liang et al., 2020; Onder et al., 2020), lymphopenia, eosinopenia, elevation of liver enzymes, escalation of lactate dehydrogenase, increase of inflammatory markers, rise of D-dimer (>1 μg/mL), elevated prothrombin time, elevation of troponin, increase in creatine phosphokinase levels, and also acute kidney injury (Guan et al., 2020). Several studies have been conducted to determine the risk groups for severe illness by SARS-CoV-2. People of any age cannot be excluded, but it is more associated with advanced age and medical comorbidities including cardiovascular disease, diabetes mellitus, hypertension, chronic lung disease, cancer, and chronic kidney disease (Zhou et al., 2020a).

Considering antihypertensive medications, there are advocates for both use and cessation of consumption. It is still unclear whether the high mortality rate of patients with hypertension comorbidity is due to the pathology of disorder or the treatment used to cure it such as ACE inhibitors and angiotensin receptor blockers (ARBs). ACE2 has been shown to be a co-receptor for viral entry and pathogenesis of SARS-CoV (Li et al., 2003). There is considerable evidence that shows the escalated expression level of ACE2 in the heart, brain, and even in urine after treatment with ARBs; however, there is limited evidence showing changes in serum or pulmonary ACE2 levels (Patel and Verma, 2020; Zhang et al., 2020c). In addition, serological, radiological, and histomorphological similarities of COVID-19-associated acute respiratory distress syndrome (ARDS) and connective tissue disease-associated interstitial lung disease (CTD-ILD) propose the postulation of triggering or simulating a form of organ-specific autoimmunity in predisposed patients. Also, some patients showed high-titer antiphospholipid antibodies, including anticardiolipin antibodies and anti-β2 glycoprotein antibodies (Zhang et al., 2020d). The immunosuppressive therapy in identified patients with autoantibodies may prevent the development of respiratory failure.

## Promising Approved Drugs and Available Clinical Trials for Prospective Treatment Options of COVID-19

With regard to treatment, immunological and pharmaceutical investigations are still ongoing. No specific therapy for COVID-19 is approved by the US Food and Drug Administration (FDA) so far, but many previously approved drugs, as an efficient approach to drug discovery named drug repurposing, are being tested on COVID-19. At the time of writing, 1,137 interventional studies have been registered in ClinicalTrials.gov (https://clinicaltrials.gov/) related to COVID-19, and this number is increasing progressively (Table S1). In particular, the ongoing clinical trials sponsored by the WHO named Solidarity will compare different treatment options, namely, remdesivir, lopinavir/ritonavir dual treatment, lopinavir/ritonavir dual treatment with interferon beta 1-alpha, and chloroquine or hydroxychloroquine (paused temporarily due to the concerns raised about the safety of the drug) against standard of care. As of May 10, 2020, more than 100 countries have been confirmed to contribute in this investigation.

**Figure 2:**
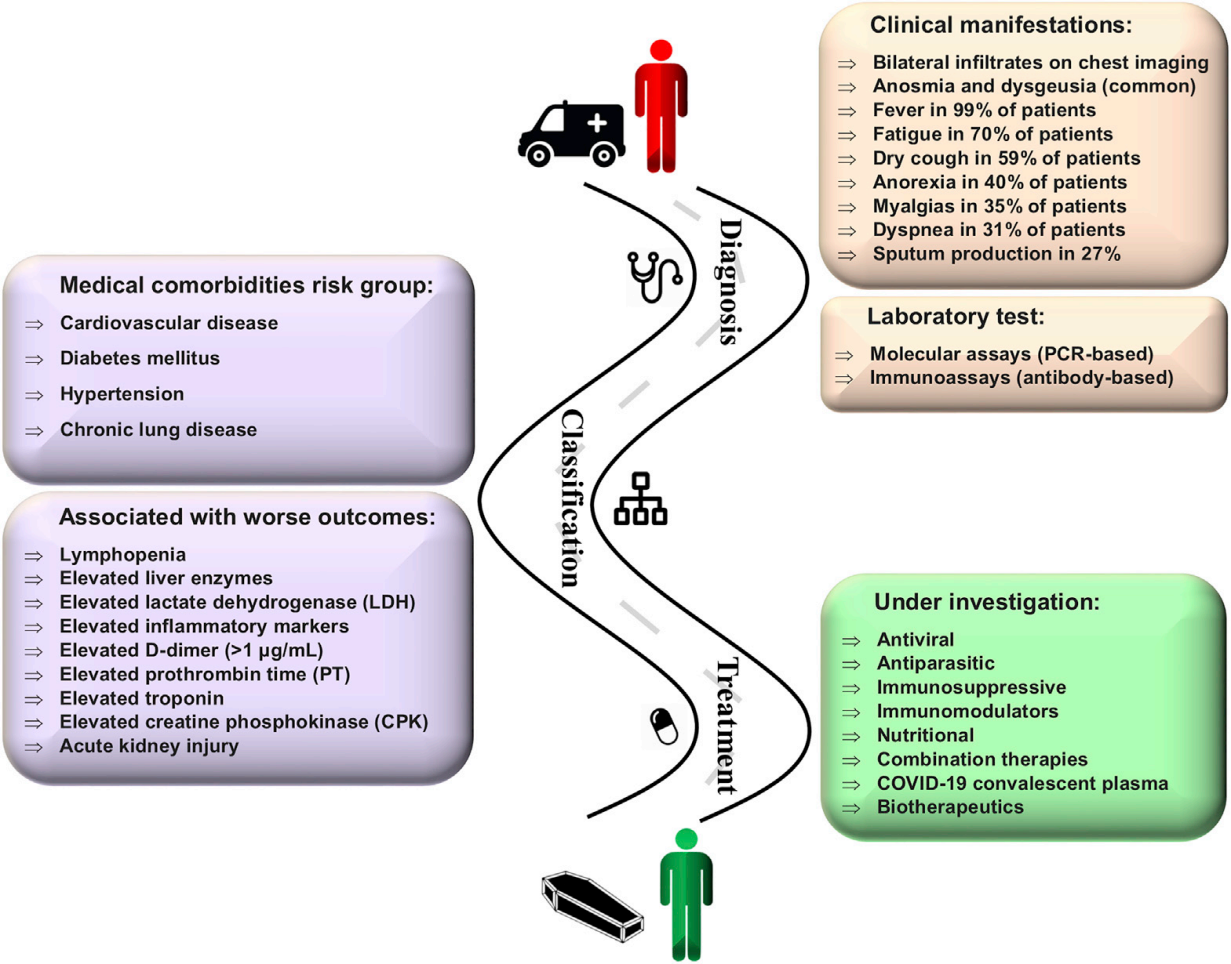
Procedure of Care for Patients with COVID-19, Including Common Symptoms, Comorbidities, Aggravating Factors, Laboratory-Based Tests, and Candidate Treatments

The interventional drugs in clinical trials can be classified based on their nature and complementary effect. In this regard, antivirals, antiparasitic drugs, immunosuppressors, immunomodulators, some well-known drugs, and nutritional drugs, in addition to combination therapies, are considered on ongoing studies for treatment, supportive care, or prevention. One can barely see the same mechanism of action inside and outside each group (Table 1), but many drugs are discovered for a specific disease and repurposed later for another disorder. Figure 2 illustrates a general path that is being traversed by clinicians these days, although the results may not be desirable in some cases due to the emergent nature of SARS-CoV-2.

**Table 1:** Properties of Repurposed Drugs for COVID-19^a^

Drug	Brand Name	Mechanism of Action	Main Indication
**Antivirals**			

Danoprevir	Ganovo	NS3/4A protease inhibitor	HCV
Favipiravir	Avigan	Nucleoside analog	Broad-spectrum antiviral
Lopinavir/ritonavir	Kaletra	Protease inhibitors Ritonavir is also a cytochrome P450 and P-gp inhibitor	HIV
Oseltamivir	Tamiflu	Inhibits viral neuraminidase and blocks the release of viral particles from host cells	Influenza
Remdesivir	–	Nucleotide analog inhibitor of RNA-dependent RNA polymerase (RdRp)	Ebola virus
Umifenovir	Arbidol	Viral fusion inhibition with the targeted membrane	Influenza, SARS-CoV

**Various well-known drugs**			

Azithromycin	Zithromax, Azithrocin, others	Inhibition translation of mRNA	Macrolide antibiotic
Carrimycin	–	Inhibition translation of mRNA	Macrolide antibiotic
Doxycycline	Doxylin, others	Inhibition bacterial protein synthesis	Tetracycline antibiotic
Chloroquine and hydroxychloroquine	Aralen, Plaquenil, Quineprox, others	Increase of lysosomal pH in antigen-presenting cells	Malaria, systemic lupus erythematosus
Nitazoxanide	Alinia, Nizonide, and others	Inhibition of the pyruvate: ferredoxin/flavodoxin oxidoreductase cycle	Broad-spectrum antiparasitic
Losartan Valsartan	Cozaar, others	Competitive angiotensin II receptor type 1 antagonist	Hypertension
Tetrandrine	–	Calcium channel blocker	Hypertension
Spironolactone	Aldactone, others	Potassium-sparing diuretic	Hypertension
Bromhexine	Bisolvon, others	Increasing lysosomal activity	Mucolytic
Dornase alfa	Pulmozyme	Recombinant human deoxyribonuclease I	Cystic fibrosis
Dexmedetomidine	Precedex	Selective alpha-2 adrenoceptor agonist	Sedation
Fluoxetine	Prozac, others	Selective serotonin reuptake inhibitor	Antidepressant

**Immunosuppressors**			

Ruxolitinib	Jakafi, Jakavi	JAK inhibition	Rheumatoid arthritis
Tocilizumab	Actemra, RoActemra	Interleukin-6 receptor antagonist	Rheumatoid arthritis
Eculizumab	Soliris	Monoclonal antibody against C5	Paroxysmal nocturnal hemoglobinuria, atypical hemolytic uremic syndrome, neuromyelitis optica
Methylprednisolone	Medrol, Meprolone, others	Inhibition of proinflammatory cytokine production	Inflammation, immune system disorders
Dexamethasone	Dexasone, Decadron, others	Inhibition of proinflammatory cytokine production	Inflammation, immune system disorders
Budesonide	Pulmicort, others	Inhibition of proinflammatory cytokine production	Asthma

**Immunomodulators**			

Camostat	Foipan	Inhibition of the transmembrane protease, serine 2 enzyme	Chronic pancreatitis
Interferons (IFN)	IFN alfa-1b (Pegasys), IFN alfa-2b (Intron-A), IFN beta-1a (Avonex, Rebif), IFN beta-1b (Betaseron, Extavia)	Initiation of JAK-STAT signaling cascades	HBV, HCV, various autoimmune disorders, and cancers
Sargramostim	Leukine	Recombinant granulocyte macrophage colony-stimulating factor	Non-Hodgkin lymphoma, acute lymphocytic leukemia
Lenalidomide	Revlimid	Induces tumor cell apoptosis	Multiple myeloma

P-gp, P-glycoprotein; HBV, hepatitis B virus; HCV, hepatitis C virus.

a Properties of repurposed drugs for COVID-19 are comprehensively described elsewhere (Lythgoe and Middleton, 2020) and are outlined briefly here.

Before clinical manifestations for COVID-19, a significant viral amplification occurs, which subsequently leads to mild symptoms. Later, at the end stage of infection, severe symptoms are mostly due to the inflammatory cell infiltration and inflammatory cytokine storm, which cause acute lung injury, ARDS, and death (Zhang et al., 2020c). Accordingly, the treatment strategy depends on the stage of the disease or a combination therapy might be considered. Also, it has been suggested that the drugs with both antiviral and anti-inflammatory properties could be taken into account for COVID-19 clinical trials including baricitinib, ruxolitinib, and fedratinib (Stebbing et al., 2020).

As examples of the most common treatment options, chloroquine and hydroxychloroquine, both antimalarial agents with anti-inflammatory and immunomodulatory activities, have shown inhibitory activity for SARS-CoV-2 similar to previous studies on SARS-CoV-1 and MERS-CoV (Sanders et al., 2020). These two treatments are being investigated for use by patients with COVID-19 based on positive *in vitro* and limited clinical data. In addition, azithromycin, a macrolide antibiotic, was found to raise the efficacy of hydroxychloroquine as a complementary therapy (Gautret et al., 2020).

Preclinical investigations proved the benefit of lopinavir/ritonavir combination therapy against COVID-19. Lopinavir is a human immunodeficiency virus type 1 (HIV-1) protease inhibitor, and in a fixed-dose combination with ritonavir, it is a potent CYP3A4 inhibitor that boosts lopinavir concentrations, could block the main protease of SARS-CoV-1, and inhibits viral replication (Ratia et al., 2008). This combination is being considered as a remedy for COVID-19, but complementary research has failed to confirm the results (Cao et al., 2020).

Remdesivir, an investigational monophosphoramidate prodrug of an adenosine analog, was developed between 2014 and 2016 in response to Ebola (Tchesnokov et al., 2019). Similar to its promising effect on SARS-CoV-1 and MERS-CoV, remdesivir has displayed potent *in vitro* activity against SARS-CoV-2 by acting as an RNA chain terminator by binding to the RNA-dependent RNA polymerase (RdRp) (Wang et al., 2020a). Although, two recent clinical trials have showed that remdesivir was not associated with dramatic clinical improvement, many clinical trials are underway (Beigel et al., 2020; Wang et al., 2020c).

Tocilizumab, a humanized monoclonal antibody, inhibits all forms of interleukin-6 (IL-6) receptors (membrane bound or soluble). Tocilizumab was first approved for rheumatoid but was later considered as a complementary treatment in cytokine-release syndrome disease (Kotch et al., 2019). Tocilizumab can diminish the effect of cytokine storming, including IL-6, which is associated with severe outcomes in patients with COVID-19 (Zhou et al., 2020a).

Several reports of clinical trials currently underway, but not yet peer-reviewed, have claimed that convalescent plasma from recovered patients displays neutralizing activity, which could be transferred to recipients by plasma infusion (Shen et al., 2020). In addition, monoclonal antibodies against the receptor-binding domain of the spike protein derived from convalescent patients have displayed neutralizing activity in a pseudovirus model and also protected against reinfection in animal models after recovery and re-challenge (Bao et al., 2020; Ju et al., 2020).

Recently, a new promising success was reported. A group of scientists claimed that human recombinant soluble ACE2 (hrsACE2) can block early stages of SARS-CoV-2 infections (Monteil et al., 2020). ACE2 seems to be a key and common receptor for many coronaviruses as it was reported to be previously critical for SARS-CoV-1 and non-critical for MERS-CoV, and now it is introduced as a crucial receptor for SARS-CoV-2 (Kuba et al., 2005; Müller et al., 2012). Accordingly, preventing the interaction of SARS-CoV-2 and ACE2 is proposed as a treatment for patients with COVID-19; however, it is not clear whether hrsACE2 can block the growth of SARS-CoV-2.

A new observation explains the international differences in COVID-19 impact by finding positive correlation between the lack of universal policies of Bacillus Calmette-Guerin (BCG) childhood vaccination (Italy, Netherlands, the United States) and the severity of COVID-19; however, this has not been confirmed by the WHO so far (WHO, 2020).

Recently, dornase alfa as a recombinant human deoxyribonuclease I is considered for clinical trials of patients with severe COVID-19 who mostly progress to an ARDS condition: hypoxemic respiratory failure associated with neutrophilia and neutrophil infiltration in the lungs, thick mucus in bronchi, and bronchiectasis (Pottecher et al., 2020). Dornase alfa acts as a mucolytic by cleaving extracellular chromosomal DNA from neutrophil extracellular traps. Accordingly, using this treatment for patients with ARDS in severe COVID-19 may lead to mucous plug clearance and accelerated recovery.

## Employment of Drug Repurposing Applications for Developing Antiviral Drugs

At present, over 40 antiviral agents are accessible for the treatment of viral infections. Unfortunately, the most commonly used antiviral drugs are accompanied by severe obstacles including insufficient selectivity, resistance, promotion of latency, toxicity, or experimental difficulties (De Clercq and Li, 2016). Drug repurposing approaches related to infectious diseases incorporate a variety of applications by harmonizing key bioinformatics and cheminformatics methods to discover a drug target that could be repurposed in the combat against a viral pathogen (Turanli et al., 2019). In addition to indisputable cost-effective benefits in the practice of drug discovery, recycled drugs gain access into clinical trials instantly or are taken up with compassionate use programs, particularly in the context of untreatable viral diseases or pandemics. Furthermore, drug repurposing provides an alternative source of data for a novel understanding of metabolic reprogramming in viral infections, as well as the organic compounds with previously unrecognized antiviral behaviors that are possible to expand in unveiling properties of virus biology (Pizzorno et al., 2019). In various instances, drug repurposing identifies formerly undiscovered biomolecular networks, transforming them into novel pharmaceutical targets, even though the determined molecules may not be implemented into clinical trials (Turanli et al., 2019b).

Over the last decade, the antiviral properties of repurposed drugs have gained considerable attention due to the lack of targeted treatments for specific emerging viruses (Law et al., 2013). Innumerable technical and bureaucratic challenges in *de novo* antiviral drug development have resulted in a very low therapeutic molecule approval rate, and as such, viral diseases such as hepatitis C and HIV are still without a vaccine. In recent years, dengue, Ebola, Zika, and coronaviruses have become public health crises, creating an ongoing demand for immediate and profitable therapeutics solutions by using antiviral drug repurposing approaches (Boldescu et al., 2017). Particularly, when an epidemic occurs, prompt actions by known drugs might help to minimize the damage until virus-specific antiviral drugs or vaccines become available. For instance, niclosamide, a well-known antihelminthic drug, proved its repurposing potential for the treatment of Zika infections (Xu et al., 2016). Another interesting candidate is chloroquine and its derivatives, which are normally indicated for malaria and have been extensively discussed in terms of their ability of the possible use for the treatment of COVID-19, as well as other diseases such as connective tissue disorders and some cancers including bladder, breast, colon, and prostate cancers (Cortegiani et al., 2020). Notably, yet there is no FDA-approved therapy for neither Ebola nor Zika virus, but promising clinical research is ongoing.

Drug repurposing methods are principally based on the analysis of biological data that make it possible to generate the hypotheses for rediscovery of available compounds for novel solutions in clinical practice and has been extensively reviewed elsewhere (Pushpakom et al., 2019). Here we summarize the most common computational drug repurposing approaches, and we detail the *in silico* drug repurposing studies of the current global health problem COVID-19.

Computational drug repurposing approaches can be broadly categorized as (1) signature based, (2) molecular docking, (3) network based, and (4) genome-wide association studies (GWAS). Signature-based drug repositioning is comparison of the pattern of gene expression profiles of a drug against that of another drug (i.e., drug-drug comparison), disease (i.e., drug-disease comparison), or clinical phenotype by using high-throughput omics data (transcriptomic, proteomic, or metabolomic), molecular structures, or adverse effect profiles (Keiser et al., 2009). Second, molecular docking is a key method in structure-based computational drug repurposing approaches based on predicting the ligand and the target complementation based on position and relative orientation of one molecule in the binding site of another molecule to generate a stable complex. Existing information of a receptor target associated with a disease enables researchers to inspect various potential drugs for a range of conditions. Reversely, libraries of compounds could be screened against receptor targets to investigate unknown interactions to discover candidates for drug repurposing process (Kitchen et al., 2004). Another key approach for computational drug design is network-based drug repurposing, which is based on the construction of the biological networks by using different data types such as gene expression patterns, protein interactions, or disease pathology (Greene and Voight, 2016). Finally, GWAS seek to determine the differences in genetic material related with common diseases and accordingly contribute a better understanding to the physiology of the diseases. The collected knowledge may also assist to define novel targets, a number of which could be allocated among diseases treated by medicine, and observable characteristics of the disease revealed by GWAS and therefore give rise to repurposing of drugs (Sanseau et al., 2012).

*In silico* drug repurposing methodologies have accelerated the studies in drug discovery through the use of data mining approaches, bioinformatics techniques, and predictive models for determining the efficacy and safety of the drugs. However, there is still a long way to reach the high success rates with repurposed drugs (e.g., repurposed candidates succeeded 9%–67%, depending on the similarity between indicated and repurposed therapeutic areas, and the novel molecular entity success rate is ∼10%) (Hay et al., 2014; Neuberger et al., 2019). This is presumably attributable to the (1) heterogeneity and incompleteness of the available data, (2) flexibility and complexity of the entire biological system and pathophysiological conditions, and (3) lack of reliable experimental data and accurate parameters related to absorption, distribution, metabolism, excretion (ADME) and toxicity, just to name a few (Brown and Patel, 2018; Ekins et al., 2007; Zhang et al., 2020a). Future studies should be directed toward obtaining a more improved test standardization and proof of concept as well as integrating the big and heterogeneous amount of data into a harmonious workflow. To leverage the benefits, drug repurposing must be performed through aforethought procedures based on the advanced stage innovation. On account of clinical trials for drug development, regardless of repositioned drug or novel molecular entity, these are expensive and time consuming, so basic science methodologies (i.e., improved animal models, accurate toxicology assessment, biomarker identification, and novel targeted delivery technologies) that undertake and reduce the possibility of hypothesis risk will be essential to increase the success rate of repurposed drugs in the product development process (Oprea et al., 2011).

## *In Silico* Drug Repurposing Studies for COVID-19

Earlier studies with other viruses of the Coronaviridae family have identified the aforementioned ACE2 receptor (Li et al., 2003) and RdRp (Imbert et al., 2006), and also coronavirus main protease (Mpro, also called 3CLpro) (Xue et al., 2008) and papain-like protease (PLpro) (Kilianski et al., 2013), as candidate drug targets. An appealing biological target for coronaviruses is the main protease by virtue of its crucial role in viral gene expression and replication. Taking this into account, Zhang et al. published the crystal structures of the Mpro of SARS-CoV-2 and docked it with a pyridone-containing α-ketoamide inhibitor. The assessment of pharmacokinetic results on mice revealed a noticeable lung tropism, which promises this lead compound as a suitable candidate for further drug development process (Zhang et al., 2020b). In addition, inhibiting the function of this enzyme blocks the viral life cycle. The absence of homologs in humans means that Mpro is an attractive non-toxic target for antiviral drug design that is not prone to off-target effects. Furthermore, virtual screening of main protease offers emergence of a powerful strategy for the rapid identification of candidate compounds from existing drugs targeting SARS-CoV-2, gaining the attention of other research teams. For instance, Jin et al. developed a data-driven drug repositioning framework, which applies both structure-based drug design and high-throughput screening approaches to inform putative drug candidates against the main protease of SARS-CoV-2. *In silico* screening followed by cell-based validation indicated that ebselen, a drug with anti-inflammatory, antioxidant, and cytoprotective activities, may be repurposed to treat COVID-19 (Jin et al., 2020). These studies audibly support the view that an antiviral compound targeting Mpro could provide a strong defense against coronavirus-associated diseases.

There are considerable opportunities in systematically investigating the dynamic interactions between host and SARS-CoV-2 to identify target human proteins for repurposing known drugs. Targeting the host-virus interactions, which are essential in the virus life cycle and demonstrate less tendency of mutational resistance, might show effectiveness in developing robust treatment options for viral infections (Kaufmann et al., 2018). However, lack of data of the pathophysiology of the SARS-CoV-2 infection restrains the development of small molecule candidates based on the host-targeted strategies. Systematically mapping interactions between SARS-CoV-2 proteins and human proteins revealed that inhibitors of mRNA translation and regulators of the Sigma1 and Sigma2 receptors might have antiviral activity (Gordon et al., 2020). Even though the role of action of compounds targeting the Sigma receptors is not well defined, receptor ligands and their mechanisms involved in immune modulator activity are considered promising antiviral targets and numerous approved compounds hold the potential to be repurposed (Gordon et al., 2020).

Characterization of viral evolution and the alterations in host cell metabolism through the use of single-cell viral metagenomic studies might shed light on the mechanisms of viral infections, including determination of the molecular signatures associated with virus-induced pathologies (Xu and Zhao, 2018). A computation-based methodology, namely, Viral-Track, applied to bronchoalveolar lavage samples from patients with severe and mild COVID-19 unveils a remarkable impact of the virus on the immune system of the patients with severe condition compared with those with mild condition, including replacement of the tissue-resident alveolar macrophages with recruited inflammatory monocytes, neutrophils, and macrophages and an altered CD8+ T cell cytotoxic response (Bost et al., 2020). Further efforts on large-scale datasets will lead to reveal many mechanistic aspects of viral infections yet to be resolved and will help to develop more efficient therapy strategies.

## Conclusion

Due to urgent need, we posit that drug repurposing is the leading method for drug discovery against COVID-19, whether through wet laboratory techniques and/or system biology approaches. Available clinical trials at both ClinicalTrials.gov (https://clinicaltrials.gov/) and WHO Solidarity includes the investigation of previously approved drugs for different indications. Taking into account that in the past two decades three coronaviruses emerged from animal reservoirs (the virus family that used to cause mild to moderate upper respiratory tract illnesses) and each one of them was cause of global concern, a common treatment may prevent coronavirus to become another lethal annual re-occurrent threat on top of seasonal influenza.

COVID-19, similar to all other pandemics throughout history, does not discriminate in terms of geopolitical borders, nationality, religion, creed, or color. A reliable forecasting of impact size and time of the ongoing COVID-19 pandemic, whether by epidemiological models or artificial intelligence-inspired methods, would be helpful in efficient management of SARS-CoV-2 repercussions. Accordingly, an international effort and unanimity are crucial to diminish the destructive effect of the COVID-19 pandemic on health care systems, casting a shadow of doubt on the futures of many nations. In spite of swift progress in virological and epidemiological knowledge of SARS-CoV-2, therapeutic challenges escalated rapidly as well. Daily changes in the number of studies and complementary results of ongoing clinical trials, including successes and failures, indicate the promising outcome in close future.

## Search Strategy and Selection Criteria

Data for this review were identified by searches of PubMed, Google Scholar, and references from relevant articles using the search terms “SARS-CoV-2,” “COVID-19,” “drug repurposing,” “antiviral therapy,” “clinical trials,” “COVID-19 therapy,” and “pandemic.” Only articles published in English were included.

## Supplemental Information

Document S1. Table S1 Ongoing COVID-19 Treatment Studies (Phase 4 and Expanded Access) Registered to Clinicaltrials.gov by June 09, 2020.

## References

[bib1] Bao L., Deng W., Gao H., Xiao C., Liu J., Xue J., Lv Q., Liu J., Yu P., Xu Y. (2020). Lack of reinfection in rhesus macaques infected with SARS-CoV-2. BioRxiv.

[bib2] Beigel J.H., Tomashek K.M., Dodd L.E., Mehta A.K., Zingman B.S., Kalil A.C., Hohmann E., Chu H.Y., Luetkemeyer A., Kline S. (2020). Remdesivir for the treatment of Covid-19 — preliminary report. N. Engl. J. Med..

[bib3] Boldescu V., Behnam M.A.M., Vasilakis N., Klein C.D. (2017). Broad-spectrum agents for flaviviral infections: dengue, Zika and beyond. Nat. Rev. Drug Discov..

[bib4] Bost P., Giladi A., Liu Y., Bendjelal Y., Xu G., David E., Blecher-Gonen R., Cohen M., Medaglia C., Li H. (2020). Host-viral infection maps reveal signatures of severe COVID-19 patients. Cell.

[bib5] Brown A.S., Patel C.J. (2018). A review of validation strategies for computational drug repositioning. Brief Bioinform..

[bib77] Cao B., Wang Y., Wen D., Liu W., Wang J., Fan G., Ruan L., Song B., Cai Y., Wei M. (2020). A trial of lopinavir–ritonavir in adults hospitalized with severe COVID-19. N. Engl. J. Med..

[bib6] Chan J.F.-W., Yuan S., Kok K.-H., To K.K.-W., Chu H., Yang J., Xing F., Liu J., Yip C.C.-Y., Poon R.W.-S. (2020). A familial cluster of pneumonia associated with the 2019 novel coronavirus indicating person-to-person transmission: a study of a family cluster. Lancet.

[bib7] Chen N., Zhou M., Dong X., Qu J., Gong F., Han Y., Qiu Y., Wang J., Liu Y., Wei Y. (2020). Epidemiological and clinical characteristics of 99 cases of 2019 novel coronavirus pneumonia in Wuhan, China: a descriptive study. Lancet.

[bib8] Chen W., Lan Y., Yuan X., Deng X., Li Y., Cai X., Li L., He R., Tan Y., Deng X. (2020). Detectable 2019-nCoV viral RNA in blood is a strong indicator for the further clinical severity. Emerging Microbes Infect..

[bib9] Cohn S., Kutalek R. (2016). Historical parallels, Ebola virus disease and cholera: understanding community distrust and social violence with epidemics. PLoS Curr..

[bib10] Cortegiani A., Ingoglia G., Ippolito M., Giarratano A., Einav S. (2020). A systematic review on the efficacy and safety of chloroquine for the treatment of COVID-19. J. Crit. Care.

[bib11] De Clercq E., Li G. (2016). Approved antiviral drugs over the past 50 years. Clin. Microbiol. Rev..

[bib12] Ekins S., Mestres J., Testa B. (2007). In silico pharmacology for drug discovery: applications to targets and beyond. Br. J. Pharmacol..

[bib13] Gautret P., Lagier J.-C., Parola P., Meddeb L., Mailhe M., Doudier B., Courjon J., Giordanengo V., Vieira V.E., Dupont H.T. (2020). Hydroxychloroquine and azithromycin as a treatment of COVID-19: results of an open-label non-randomized clinical trial. Int. J. Antimicrob. Agents.

[bib14] Giacomelli A., Pezzati L., Conti F., Bernacchia D., Siano M., Oreni L., Rusconi S., Gervasoni C., Ridolfo A.L., Rizzardini G. (2020). Self-reported olfactory and taste disorders in patients with severe acute respiratory coronavirus 2 infection: a cross-sectional study. Clin. Infect. Dis..

[bib15] Gorbalenya A.E. (2020). Severe acute respiratory syndrome-related coronavirus–The species and its viruses, a statement of the Coronavirus Study Group. BioRxiv.

[bib16] Gordon D.E., Jang G.M., Bouhaddou M., Xu J., Obernier K., White K.M., O’Meara M.J., Rezelj V.V., Guo J.Z., Swaney D.L. (2020). A SARS-CoV-2 protein interaction map reveals targets for drug repurposing. Nature.

[bib17] Greene C.S., Voight B.F. (2016). Pathway and network-based strategies to translate genetic discoveries into effective therapies. Hum. Mol. Genet..

[bib18] Guan W.-j., Ni Z.-y., Hu Y., Liang W.-H., Ou C.-Q., He J.-X., Liu L., Shan H., Lei C.-l., Hui D.S. (2020). Clinical characteristics of coronavirus disease 2019 in China. New Engl. J. Med..

[bib19] Hay M., Thomas D.W., Craighead J.L., Economides C., Rosenthal J. (2014). Clinical development success rates for investigational drugs. Nat. Biotechnol..

[bib20] Huang C., Wang Y., Li X., Ren L., Zhao J., Hu Y., Zhang L., Fan G., Xu J., Gu X. (2020). Clinical features of patients infected with 2019 novel coronavirus in Wuhan, China. Lancet.

[bib21] Imbert I., Guillemot J.-C., Bourhis J.-M., Bussetta C., Coutard B., Egloff M.-P., Ferron F., Gorbalenya A.E., Canard B. (2006). A second, non-canonical RNA-dependent RNA polymerase in SARS coronavirus. EMBO J..

[bib22] Jin Z., Du X., Xu Y., Deng Y., Liu M., Zhao Y., Zhang B., Li X., Zhang L., Peng C. (2020). Structure of Mpro from COVID-19 virus and discovery of its inhibitors. Nature.

[bib23] Ju B., Zhang Q., Ge X., Wang R., Yu J., Shan S., Zhou B., Song S., Tang X., Yu J. (2020). Potent human neutralizing antibodies elicited by SARS-CoV-2 infection. Nature.

[bib24] Kaufmann S.H.E., Dorhoi A., Hotchkiss R.S., Bartenschlager R. (2018). Host-directed therapies for bacterial and viral infections. Nat. Rev. Drug Discov..

[bib25] Keiser M.J., Setola V., Irwin J.J., Laggner C., Abbas A.I., Hufeisen S.J., Jensen N.H., Kuijer M.B., Matos R.C., Tran T.B. (2009). Predicting new molecular targets for known drugs. Nature.

[bib26] Kilianski A., Mielech A.M., Deng X., Baker S.C. (2013). Assessing activity and inhibition of Middle East respiratory syndrome coronavirus papain-like and 3C-like proteases using luciferase-based biosensors. J. Virol..

[bib27] Kitchen D.B., Decornez H., Furr J.R., Bajorath J. (2004). Docking and scoring in virtual screening for drug discovery: methods and applications. Nat. Rev. Drug Discov..

[bib28] Kotch C., Barrett D., Teachey D.T. (2019). Tocilizumab for the treatment of chimeric antigen receptor T cell-induced cytokine release syndrome. Expert Rev. Clin. Immunol..

[bib29] Kuba K., Imai Y., Rao S., Gao H., Guo F., Guan B., Huan Y., Yang P., Zhang Y., Deng W. (2005). A crucial role of angiotensin converting enzyme 2 (ACE2) in SARS coronavirus–induced lung injury. Nat. Med..

[bib30] Law G.L., Tisoncik-Go J., Korth M.J., Katze M.G. (2013). Drug repurposing: a better approach for infectious disease drug discovery?. Curr. Opin. Immunol..

[bib31] Li W., Moore M.J., Vasilieva N., Sui J., Wong S.K., Berne M.A., Somasundaran M., Sullivan J.L., Luzuriaga K., Greenough T.C. (2003). Angiotensin-converting enzyme 2 is a functional receptor for the SARS coronavirus. Nature.

[bib32] Li W., Shi Z., Yu M., Ren W., Smith C., Epstein J.H., Wang H., Crameri G., Hu Z., Zhang H. (2005). Bats are natural reservoirs of SARS-like coronaviruses. Science.

[bib33] Liang W., Guan W., Chen R., Wang W., Li J., Xu K., Li C., Ai Q., Lu W., Liang H. (2020). Cancer patients in SARS-CoV-2 infection: a nationwide analysis in China. Lancet Oncol..

[bib34] Liu Y., Gayle A.A., Wilder-Smith A., Rocklöv J. (2020). The reproductive number of COVID-19 is higher compared to SARS coronavirus. J. Trav. Med..

[bib35] Lu R., Zhao X., Li J., Niu P., Yang B., Wu H., Wang W., Song H., Huang B., Zhu N. (2020). Genomic characterisation and epidemiology of 2019 novel coronavirus: implications for virus origins and receptor binding. The Lancet.

[bib36] Lythgoe M.P., Middleton P. (2020). Ongoing clinical trials for the management of the COVID-19 pandemic. Trends Pharmacol. Sci..

[bib37] Monteil V., Kwon H., Prado P., Hagelkru?ys A., Wimmer R.A., Stahl M., Leopoldi A., Garreta E., Hurtado del Pozo C., Prosper F. (2020). Inhibition of SARS-CoV-2 infections in engineered human tissues using clinical-grade soluble human ACE2. Cell.

[bib38] Müller M.A., Raj V.S., Muth D., Meyer B., Kallies S., Smits S.L., Wollny R., Bestebroer T.M., Specht S., Suliman T. (2012). Human coronavirus EMC does not require the SARS-coronavirus receptor and maintains broad replicative capability in mammalian cell lines. MBio.

[bib39] Neuberger A., Oraiopoulos N., Drakeman D.L. (2019). Renovation as innovation: is repurposing the future of drug discovery research?. Drug Discov. Today.

[bib40] Onder G., Rezza G., Brusaferro S. (2020). Case-fatality rate and characteristics of patients dying in relation to COVID-19 in Italy. JAMA.

[bib41] Oprea T.I., Bauman J.E., Bologa C.G., Buranda T., Chigaev A., Edwards B.S., Jarvik J.W., Gresham H.D., Haynes M.K., Hjelle B. (2011). Drug repurposing from an academic perspective. Drug Discov. Today Ther. Strateg..

[bib42] Patel A.B., Verma A. (2020). COVID-19 and angiotensin-converting enzyme inhibitors and angiotensin receptor blockers: what is the evidence?. JAMA.

[bib43] Pechous R.D., Sivaraman V., Stasulli N.M., Goldman W.E. (2016). Pneumonic plague: the darker side of Yersinia pestis. Trends Microbiol..

[bib44] Pizzorno A., Padey B., Terrier O., Rosa-Calatrava M. (2019). Drug repurposing approaches for the treatment of influenza viral infection: reviving old drugs to fight against a long-lived enemy. Front. Immunol..

[bib45] Pottecher J., Noll E., Borel M., Audibert G., Gette S., Meyer C., Gaertner E., Legros V., Carapito R., Uring-Lambert B. (2020). Protocol for TRAUMADORNASE: a prospective, randomized, multicentre, double-blinded, placebo-controlled clinical trial of aerosolized dornase alfa to reduce the incidence of moderate-to-severe hypoxaemia in ventilated trauma patients. Trials.

[bib46] Pushpakom S., Iorio F., Eyers P.A., Escott K.J., Hopper S., Wells A., Doig A., Guilliams T., Latimer J., McNamee C. (2019). Drug repurposing: progress, challenges and recommendations. Nat. Rev. Drug Discov..

[bib47] Ratia K., Pegan S., Takayama J., Sleeman K., Coughlin M., Baliji S., Chaudhuri R., Fu W., Prabhakar B.S., Johnson M.E. (2008). A noncovalent class of papain-like protease/deubiquitinase inhibitors blocks SARS virus replication. Proc. Natl. Acad. Sci. U S A.

[bib48] Rothe C., Schunk M., Sothmann P., Bretzel G., Froeschl G., Wallrauch C., Zimmer T., Thiel V., Janke C., Guggemos W. (2020). Transmission of 2019-nCoV infection from an asymptomatic contact in Germany. New Engl. J. Med..

[bib49] Sanders J.M., Monogue M.L., Jodlowski T.Z., Cutrell J.B. (2020). Pharmacologic treatments for coronavirus disease 2019 (COVID-19): a review. JAMA.

[bib50] Sanseau P., Agarwal P., Barnes M.R., Pastinen T., Richards J.B., Cardon L.R., Mooser V. (2012). Use of genome-wide association studies for drug repositioning. Nat. Biotechnol..

[bib51] Shen C., Wang Z., Zhao F., Yang Y., Li J., Yuan J., Wang F., Li D., Yang M., Xing L. (2020). Treatment of 5 critically ill patients with COVID-19 with convalescent plasma. JAMA.

[bib52] Simonsen L., Chowell G., Andreasen V., Gaffey R., Barry J., Olson D., Viboud C. (2018). A review of the 1918 herald pandemic wave: importance for contemporary pandemic response strategies. Ann. Epidemiol..

[bib54] Stebbing J., Phelan A., Griffin I., Tucker C., Oechsle O., Smith D., Richardson P. (2020). COVID-19: combining antiviral and anti-inflammatory treatments. Lancet Infect. Dis..

[bib55] Tang A., Tong Z., Wang H., Dai Y., Li K., Liu J., Wu W., Yuan C., Yu M., Li P. (2020). Detection of novel coronavirus by RT-PCR in stool specimen from asymptomatic child, China. Emerging Infect. Dis..

[bib56] Tang X., Wu C., Li X., Song Y., Yao X., Wu X., Duan Y., Zhang H., Wang Y., Qian Z. (2020). On the origin and continuing evolution of SARS-CoV-2. Natl. Sci. Rev..

[bib57] Tchesnokov E.P., Feng J.Y., Porter D.P., Götte M. (2019). Mechanism of inhibition of Ebola virus RNA-dependent RNA polymerase by remdesivir. Viruses.

[bib58] Turanli B., Altay O., Borén J., Turkez H., Nielsen J., Uhlen M., Arga K.Y., Mardinoglu A. (2019). Systems biology based drug repositioning for development of cancer therapy. Semin. Cancer Biol..

[bib59] Turanli B., Zhang C., Kim W., Benfeitas R., Uhlen M., Arga K.Y., Mardinoglu A. (2019). Discovery of therapeutic agents for prostate cancer using genome-scale metabolic modeling and drug repositioning. EBioMedicine.

[bib60] Wang M., Cao R., Zhang L., Yang X., Liu J., Xu M., Shi Z., Hu Z., Zhong W., Xiao G. (2020). Remdesivir and chloroquine effectively inhibit the recently emerged novel coronavirus (2019-nCoV) in vitro. Cell Res..

[bib61] Wang Y., Wang Y., Chen Y., Qin Q. (2020). Unique epidemiological and clinical features of the emerging 2019 novel coronavirus pneumonia (COVID-19) implicate special control measures. J. Med. Virol..

[bib62] Wang Y., Zhang D., Du G., Du R., Zhao J., Jin Y., Fu S., Gao L., Cheng Z., Lu Q. (2020). Remdesivir in adults with severe COVID-19: a randomised, double-blind, placebo-controlled, multicentre trial. Lancet.

[bib63] WHO (2020). Bacille Calmette-Guérin (BCG) Vaccination and COVID-19: Scientific Brief, 12 April 2020.

[bib64] Xiao S.Y., Wu Y., Liu H. (2020). Evolving status of the 2019 novel coronavirus Infection: proposal of conventional serologic assays for disease diagnosis and infection monitoring [Commentary/Review]. J. Med. Virol..

[bib65] Xu M., Lee E.M., Wen Z., Cheng Y., Huang W.K., Qian X., Tcw J., Kouznetsova J., Ogden S.C., Hammack C. (2016). Identification of small-molecule inhibitors of Zika virus infection and induced neural cell death via a drug repurposing screen. Nat. Med..

[bib66] Xu Y., Zhao F. (2018). Single-cell metagenomics: challenges and applications. Protein Cell.

[bib67] Xue X., Yu H., Yang H., Xue F., Wu Z., Shen W., Li J., Zhou Z., Ding Y., Zhao Q. (2008). Structures of two coronavirus main proteases: implications for substrate binding and antiviral drug design. J. Virol..

[bib68] Yu P., Zhu J., Zhang Z., Han Y., Huang L. (2020). A familial cluster of infection associated with the 2019 novel coronavirus indicating potential person-to-person transmission during the incubation period. J. Infect. Dis..

[bib69] Zhang J.D., Sach-Peltason L., Kramer C., Wang K., Ebeling M. (2020). Multiscale modelling of drug mechanism and safety. Drug Discov. Today.

[bib70] Zhang L., Lin D., Sun X., Curth U., Drosten C., Sauerhering L., Becker S., Rox K., Hilgenfeld R. (2020). Crystal structure of SARS-CoV-2 main protease provides a basis for design of improved α-ketoamide inhibitors. Science.

[bib71] Zhang W., Zhao Y., Zhang F., Wang Q., Li T., Liu Z., Wang J., Qin Y., Zhang X., Yan X. (2020). The use of anti-inflammatory drugs in the treatment of people with severe coronavirus disease 2019 (COVID-19): the experience of clinical immunologists from China. Clin. Immunol..

[bib72] Zhang Y., Xiao M., Zhang S., Xia P., Cao W., Jiang W., Chen H., Ding X., Zhao H., Zhang H. (2020). Coagulopathy and antiphospholipid antibodies in patients with Covid-19. N. Engl. J. Med..

[bib73] Zhou F., Yu T., Du R., Fan G., Liu Y., Liu Z., Xiang J., Wang Y., Song B., Gu X. (2020). Clinical course and risk factors for mortality of adult inpatients with COVID-19 in Wuhan, China: a retrospective cohort study. Lancet.

[bib74] Zhou P., Yang X.-L., Wang X.-G., Hu B., Zhang L., Zhang W., Si H.-R., Zhu Y., Li B., Huang C.-L. (2020). A pneumonia outbreak associated with a new coronavirus of probable bat origin. Nature.

[bib75] Zhu N., Zhang D., Wang W., Li X., Yang B., Song J., Zhao X., Huang B., Shi W., Lu R. (2020). A novel coronavirus from patients with pneumonia in China, 2019. N. Engl. J. Med..

[bib76] Zou L., Ruan F., Huang M., Liang L., Huang H., Hong Z., Yu J., Kang M., Song Y., Xia J. (2020). SARS-CoV-2 viral load in upper respiratory specimens of infected patients. N. Engl. J. Med..

